# A Chaplain-led Spiritual Life Review Pilot Study for Patients with Brain Cancers and Other Degenerative Neurologic Diseases

**DOI:** 10.5041/RMMJ.10199

**Published:** 2015-04-29

**Authors:** Katherine M. Piderman, Carmen Radecki Breitkopf, Sarah M. Jenkins, Terin T. Euerle, Laura A. Lovejoy, Gracia M. Kwete, Aminah Jatoi

**Affiliations:** 1 Coordinator of Research, Chaplain Services; Assistant Professor of Psychiatry, Mayo Clinic, Rochester, MN, USA;; 2 Associate Professor, Department of Health Services Research, Mayo Clinic, Rochester, MN, USA;; 3 Statistician III, Biostatistics and Informatics, Mayo Clinic, Rochester, MN, USA;; 4 Mayo Medical Student, Mayo Medical School, Mayo Clinic, Rochester, MN, USA;; 5 Staff Chaplain, Chaplain Services, Mayo Clinic, Rochester, MN, USA and; 6 Consultant, Medical Oncology; Professor of Oncology, Mayo Clinic, Rochester, MN, USA

**Keywords:** Brain cancer, chaplain, life review, neurodegenerative disease, resiliency, spirituality

## Abstract

**Objective:**

This pilot study was designed to describe changes in spiritual well-being (SWB), spiritual coping, and quality of life (QOL) in patients with brain cancer or other neurodegenerative diseases participating in a chaplain-led spiritual life review interview and development of a spiritual legacy document (SLD).

**Methods:**

Eligible participants were enrolled and completed baseline questionnaires. They were interviewed by a board-certified chaplain about spiritual influences, beliefs, practices, values, and spiritual struggles. An SLD was prepared for each participant, and one month follow-up questionnaires were completed. Two cases are summarized, and spiritual development themes are illustrated within a spiritual development framework.

**Results:**

A total of 27 patients completed baseline questionnaires and the interview; 24 completed the SLD, and 15 completed the follow-up questionnaire. Increases in SWB, religious coping, and QOL were detected. The majority maintained the highest (best) scores of negative religious coping, demonstrating minimal spiritual struggle.

**Conclusions:**

Despite the challenges of brain cancers and other neurodegenerative diseases, participants demonstrated improvements in SWB, positive religious coping, and QOL. Patient comments indicate that benefit is related to the opportunity to reflect on and integrate spiritual experiences and to preserve them for others. Research with a larger, more diverse sample is needed, as well as clinical applications for those too vulnerable to participate in longitudinal follow-up.

## INTRODUCTION

The experience of serious illness often provokes an unexpected and unwelcome encounter with vulnerability and ultimate mortality. Such experiences invite, and even demand, reflection on established beliefs, patterns of behavior and relationships, and a recalculation of priorities. While conventional medical interventions are essential to the well-being of patients, attention to these less visible but very real issues is also important.^1^,^2^ For many with serious illnesses,^3^–^6^ including those with brain tumors and other neurodegenerative diseases,^7^,^8^ spirituality has a key role. Definitions of spirituality tend to point back to the Latin root “spiritus” or breath. Spirituality, then, is the breath of life, not simply physical life, but vitality and meaning. Often, spirituality also involves a relationship with the Sacred or a truth beyond one’s self.^9^,^10^

There is evidence that the sharing of a personal spiritual life-story can be therapeutic for patients and also instructive for clinicians, especially those committed to providing respectful, holistic care.^11^–^13^ Many medical facilities include on their staff professional chaplains who have been endorsed by a particular religious denomination and fulfilled the theological and clinical requirements of an endorsing body, e.g. the Association of Professional Chaplains, the National Association of Catholic Chaplains, the National Association of Jewish Chaplains, to provide spiritual assessment, spiritual companionship, and spiritual care to patients as they grapple with illness.^5^,^14^ Though spirituality has been linked to many health outcomes,^15^ there is a need for research on the efficacy of the ministry that chaplains provide.^16^ This paper describes the background and preliminary results of a pilot study for patients with brain cancers and other neuro-degenerative diseases participating in a chaplain-led spiritual legacy process. Patients with these conditions were chosen for the pilot study because of their vulnerability to cognitive decline, communication difficulties, and, in some cases, a shortened lifespan. It seemed important to give them an opportunity to document important issues related to their spirituality while they were able. The aim of the study was to describe changes in the spiritual well-being (SWB), spiritual coping, and quality of life (QOL) of those participating in a chaplain-led interview and developing a unique spiritual legacy document (SLD).

This paper also presents a summary of the interviews of two participants. Themes of spiritual development within the interview are illustrated using a model developed by Pargament and colleagues.^17^ This model describes three constructs inherent in spiritual development: *discovery*, *conservation*, and *transformation*. Discovery involves a process of spiritual formation, usually during childhood, that occurs by engaging in and learning from spiritual relationships and experiences. Conservation involves maintaining one’s learned spirituality in ways that are coherent with life roles and meaning. Transformation involves a re-examination of one’s spirituality, often in the context of spiritual struggle, leading to new or deeper convictions.

## METHODS

This study was approved by the Mayo Clinic Institutional Review Board in 2012. Participants were at least 18 years old, with brain cancer or neurodegenerative illnesses. Excluded were those who were psychiatric inpatients or those thought to be at risk of inflicting self-harm or harm to others. Previous spiritual care from a chaplain at Mayo Clinic was not specified as inclusion or exclusion criteria. Enrollment took place through the Mayo Clinic Cancer Center, but self-referrals were also accepted.

Following the introduction of the study and informed consent procedures, patients supplied demographic information and completed baseline questionnaires: the Functional Assessment of Chronic Illness Therapy–SWB (FACIT-Sp-12),^18^ Brief Religious Coping Scale (Brief RCOPE),^19^,^20^ and the QOL Linear Analog Self-Assessment (LASA).^21^,^22^ Then, each patient was interviewed by a board-certified chaplain using an interview guide adapted from the FICA Spiritual Assessment Tool^5^,^6^ and Dignity Therapy.^23^ Questions were related to early spiritual influences, beliefs, practices, values, and spiritual struggle.^24^ The length of the interview was not predetermined but was kept flexible to accommodate the varied expressive ability, fatigue level, and engagement of the patients with the questions and the interviewer. The interview was audio-recorded and transcribed, and a draft of a unique SLD was prepared for each patient. After the content was edited by members of the research team and approved by the patient, it was professionally printed in an 8 × 8 inch spiral-bound booklet, and they were able to order up to 25 copies free of charge. Follow-up questionnaires, which were identical to the admission questionnaires, were mailed approximately one month after receipt of SLD.

### Analysis

Patients were divided into three disease groups: those with advanced brain cancers, those with early-stage brain cancers, and those with non-cancerous neurodegenerative diseases. Continuous data were summarized with means and standard deviations, and categorical data with frequencies and percentages. Then, the percentage of participants in each group who improved from baseline to follow-up and the percentage of patients who maintained a maximum possible score from baseline were summarized.

## RESULTS

Thirty-two patients enrolled in this study; 5 withdrew, and 27 completed baseline questionnaires and an interview with a chaplain. The ages of the 27 participants ranged from 21.8 to 77.1 years (average 52.0). Approximately half (15; 55.6%) were female. Most were currently married (19; 70.4%) and had at least a four year college degree (17; 63.0%). Half (14; 51.9%) were Protestant, six (22.2%) were Catholic, one (3.7%) was Muslim, and six (22.2%) indicated no religious preference. Twelve patients (44.4%) had advanced brain cancers, nine (33.3%) had early-stage brain cancers, and six (22.2%) had non-cancerous neurodegenerative diseases. Only two of the 27 patients interviewed had a previous professional relationship with the interviewing chaplain. The length of the interviews ranged from approximately 30 minutes to 2.5 hours. Four patients were interviewed in two separate sessions, one because of fatigue and three because of their desire to add substantial material to their document. Twenty-four patients completed their SLD, and 15 of these completed the follow-up questionnaire. Those who did not included 10 patients who died prior to follow-up and two who did not respond to contact attempts.

In several cases, patients scored the highest possible on baseline measures reflecting SWB, religious coping, and QOL, but some improvement was detected in all disease groups. Most notably, 100% (4/4) of those with neurodegenerative diseases reported an increase in SWB on the FACITSp-12 and in positive religious coping. Additionally, 75% (3/4) of this group improved on the SWB LASA and on the FACIT-Sp-12’s meaning/peace subscale. Of those with advanced brain tumors, 66.7% (2/3) reported an increase in positive religious coping and overall QOL, while 75% (6/8) of those with early-stage brain cancers reported improved positive religious coping, and 62.5% (5/8) reported improved SWB on the FACIT-Sp-12 and its meaning/peace subscale. The majority of patients in each disease group maintained the highest possible score on the negative religious coping subscale, with higher scores indicating less spiritual distress, i.e. 66% (2/3) with advanced brain tumors, 50% (4/8) with early-stage brain tumors, and 75% (3/4) with other neurologic illnesses. The score of one patient with an early-stage brain tumor improved on this measure. No statistical testing was done because of the small number per group (Table 1).

**Table 1.** Improvement from Baseline to First Follow-up within Scale of Interest among Patients Who Have at Least One Follow-up Available by Disease Group.

**Scale of Interest**	**Advanced Brain Tumor (*n*=3)**	**Early-stage Brain Tumor (*n*=8)**	**Other Neurological Disease (*n*=4)**
**FACIT-Sp-12**			
Did not improve	2 (66.7%)	3 (37.5%)	0 (0.0%)
Improved from baseline	1 (33.3%)	5 (62.5%)	4 (100.0%)
**FACIT-Sp-12: meaning/peace subscale**			
Did not improve	2 (66.7%)	2 (25.0%)	1 (25.0%)
Maintained highest possible from baseline	0 (0.0%)	1 (12.5%)	0 (0.0%)
Improved from baseline	1 (33.3%)	5 (62.5%)	3 (75.0%)
**FACIT-Sp-12: faith subscale**			
Did not improve	1 (33.3%)	4 (50.0%)	2 (50.0%)
Maintained highest possible from baseline	2 (66.7%)	3 (37.5%)	0 (0.0%)
Improved from baseline	0 (0.0%)	1 (12.5%)	2 (50.0%)
**LASA: Your overall quality of life**			
Did not improve	1 (33.3%)	6 (75.0%)	2 (50.0%)
Improved from baseline	2 (66.7%)	2 (25.0%)	2 (50.0%)
**LASA: Your overall spiritual well-being**			
Did not improve	2 (66.7%)	4 (50.0%)	1 (25.0%)
Maintained highest possible from baseline	0 (0.0%)	1 (12.5%)	0 (0.0%)
Improved from baseline	1 (33.3%)	3 (37.5%)	3 (75.0%)
**Brief RCOPE positive religious coping subscale**			
Did not improve	1 (33.3%)	2 (25.0%)	0 (0.0%)
Improved from baseline	2 (66.7%)	6 (75.0%)	4 (100.0%)
**Brief RCOPE negative religious coping subscale**			
Did not improve	1 (33.3%)	3 (37.5%)	1 (25.0%)
Maintained highest possible from baseline	2 (66.7%)	4 (50.0%)	3 (75.0%)
Improved from baseline	0 (0.0%)	1 (12.5%)	0 (0.0%)

FACIT-Sp-12, functional assessment of chronic illness therapy–SWB; LASA, linear analog self-assessment; Brief RCOPE, brief religious coping scale.

Summaries of the spiritual legacy interview transcripts of two patients, identified by pseudonyms, are presented below. These patients were similar in several ways. Both were married men in their mid-60s with adult children, both had been previously employed as engineers, and both had been diagnosed with an advanced brain tumor and died on study. Their spiritual perspectives were very different; one was Roman Catholic and the other an agnostic, but both demonstrate a process of spiritual development that is coherent with Pargament’s model described briefly above.

### Case 1

Mr Wheeler was raised on a farm in the mid-western United States. He *discovered* his faith through the influence of his parents and extended family who were devout Roman Catholics. Mr Wheeler attended worship services at least weekly and participated in regular daily prayer with his family. All were activities he treasured. Mr Wheeler credited his parochial school teachers with having a profound influence on him spiritually. They helped him to move towards *conservation* of his beliefs and making them his own. Activities of spiritual *conservation* continued after Mr Wheeler became an adult, married, and had children. He and his wife were active in their church and continued to take adult religion classes and support their children in their religious formation.

*Transformation* occurred when Mr Wheeler’s son became ill at age 5 and died shortly afterwards. Mr Wheeler mourned deeply. He was “sad and depressed for seven years.” His son had made a craft at Sunday school shortly before he died with this refrain from Psalm 136 on it: “God’s love is everlasting.” Mr Wheeler valued it as a message from their son to trust in God, but in his grief he wrestled with its veracity and meaning. Ultimately, Mr Wheeler found that his son’s death had “strengthened” his faith, and one day he “just decided to be happy,” and he was able to move forward. His beliefs were not radically different, but were now informed by his suffering. He found music, including church music, to be a source of consolation for him and a way that he could express his faith. He learned to play the guitar and became active in a men’s choir at his church. Then he described buying “a round table” for the dining room. He had come to a new sense of interconnectedness with his wife and daughters and did not want a position at the head of the dinner table. He said, “A round table fits round prayers,” meaning that he wanted to be sure that his wife and daughters felt equal to him and respected in their family prayer and conversation.

Mr Wheeler’s brain tumor brought new challenges for him. He described his thinking as “not so good” and like being in “a weed patch.” Unfortunately, music, including his own singing, gave him a headache, and he lost that source of consolation. Most significantly, Mr Wheeler faced an abrupt confrontation with his mortality. *Transformation* was demonstrated again not in a change in his belief system, but a deeper reliance on it. Psalm 27, which speaks of trusting in God during times of adversity, had been one of Mr Wheeler’s favorite scripture passages, and it became more meaningful to him as he dealt with anger and sadness related to his diagnosis.

Always a kind and sensitive man, he felt an imperative to express his gratitude, love, and important values to his family. He told them: “I gotta sit down with you folks and one by one, I have to thank you … for doing what you are doing.” He reported that he had accomplished this before he died. Mr Wheeler requested that the following statements be included in his SLD: “I’d like my family and others who love me to know that these two sayings have been my mottos: ‘Forgive one another.’ and ‘Be a person of integrity.’ I’d also like to leave them with three more words, ‘I love you’.”

### Case 2

Mr Nicholson credited his father and grandfather as having the most influence on him spiritually. He said, “They slanted me towards agnostic.” He was aware of the Ten Commandments as a code of conduct, but said, “That’s as close as I got to religion.” As he grew up, he *conserved* this approach to spirituality, summarized by this statement, “I don’t believe in religion.” He was greatly influenced by his education and stated that he put his faith in science because of its predictability. Then, as an adult, he put his trust in his wife, because she, like science, was “predictable” and their relationship “enriched” him. Apart from his wife, Mr Nicholson led a rather insular life without tolerance for mystery and for those who thought differently than he did.

As a young adult, Mr Nicholson struggled with alcoholism and a negative and critical approach to life. He could be very judgmental when things or people were not as he expected them to be. His decision to quit drinking alcohol “made a huge difference,” especially in terms of his relationships. If he ever got “off track” he said, “Just looking back was enough,” to turn him around.

Mr Nicholson’s diagnosis of brain cancer prompted a bounty of unexpected support and help from others in his life. This caused him to reflect and prompted *transformation* in his spiritual world view and approach to others. His eyes were opened to the goodness of others and he experienced a sense of gratitude and connectedness that was new to him. He also found himself more aware of others in general and “more empathetic to people who have life-threatening diseases.” He was aware of the harsh way he had treated others when he was drinking and, in later years, under stress. He expressed his deep remorse for the “devastating” consequences related to this behavior. While his agnostic perspective led him “not to expect much” as he looked to the future, he began to entertain the possibility of an afterlife for himself. If there was, he planned to do what he could to help his family whenever they needed him. He said, “I’ll leave indicators that I got the message and that I’m operating.” He advised his loved ones, “Be good to people … The act of sharing is pretty important. Make sure you do that throughout your life.”

## DISCUSSION

This study provided patients with brain cancers and other neurodegenerative diseases an opportunity to give voice to their spirituality in a comprehensive manner within the context of a relationship with a spiritual care professional, and to prepare an SLD for their loved ones. Though participants had a variety of belief systems and were in the midst of challenging disease processes, enrollment data demonstrated that most patients had relatively high levels of spiritual well-being and quality of life, and relatively low levels of spiritual distress. Follow-up data indicated that most patients either maintained or improved in these areas. This seems quite remarkable and encourages further exploration of the potential for spiritual well-being and spiritual growth during times of medical adversity.

This study is limited by its small sample size and the lack of a control group, so definite conclusions based on its results must be curbed. However, spontaneous comments by patients, both within the interview and afterwards, suggest that their experience in the study was helpful to them in several ways. Specifically, patients expressed their gratitude for the opportunity to reflect on their lives and to integrate both positive and challenging experiences related to their diagnosis and disease progression. Patients also reported valuing the opportunity to preserve significant aspects of their spiritual journey for their loved ones and others, and hoped that their words would influence them in positive ways. Several also offered their appreciation for the respectful and “careful listening” of the interviewer. These comments alongside the survey results suggest that the spiritual life review process benefitted patients by affirming their dignity and offering them an opportunity to make a difference in the lives of others.^23^,^25^ These experiences could certainly be related to the improvements and stability of the survey data. Additionally, this study’s emphasis on spirituality and the opportunity to explore spiritual issues from all stages of life with a spiritual care professional may have contributed as well (Table 2).

**Table 2.** A Selection of Patients’ Spontaneous Comments about their Participation in the Study.

Patient	Comment
1	I was hesitant at first, but once I got into it I really have enjoyed it. It has been great to put things into words for myself and to know that my family will have this. But also, it means a lot that I might be able to help people know what’s important. You never know when something like this might happen and I’d like them to know that there’s no time to waste not living like you really want to live.
2	Thank you so much for the wonderful experience of seeing more clearly how God has blessed me. You have put the music to my story. My spiritual legacy … is bringing out such beautiful comments from family and friends. I feel myself that people like it so much because they know it is real … I think everyone regardless of health situation should ponder those questions. They make it very clear how we are continually blessed and aided by God.
3	Thank you for all your help with my Spiritual Legacy Journal. I really enjoyed the process as I had to speak and read the words of my faith journey. I came home from our meeting last week and got out my “Funeral File.” I have been compiling additional thoughts as I plan to give a copy of this document to my children and siblings. It will be nice to have so much about me in one location so when those difficult decisions need to be made, I can have some suggestions ready for them.
4	Thank you for your kind and sensitive listening. The most interesting and useful question for me was about my greatest achievement. It gives me a new perspective on the importance of paying attention to what is going on in my life, and responding to it with compassion and courage.
5	Honestly, I think being here and speaking about the change in me [since diagnosis] is the best thing I’ve done [in my life] so far … I felt the peacefulness that [talking about] it has brought into my life. So probably if someone reads or listens to what I’m saying, it might ring a bell somewhere. It might be a wake-up call for others.

As in any clinical study, it is very important to be mindful of those who did not improve on various measures and to explore reasons for this. Disease progression is a likely contributor, but it is also possible that other adversity occurred. No participants indicated that the study itself was unsettling in any way. It will be important to include a targeted evaluation of the participants’ experience to future protocols. It is also important to understand the relationship of SWB, QOL, and positive religious coping with spiritual struggle. In the results of this study, it appears that doing well spiritually does not preclude spiritual struggle. This needs to be verified, as it could be very helpful information for all who seek to be attentive to a patient’s spirituality.

The two summarized cases demonstrate the feasibility of using Pargament’s framework to identify experiences related to spiritual development. The investigators intend to use this model in their qualitative analysis of the interview data. The cases also highlight specific ways that adversity challenged the patient’s spiritual perspective and promoted spiritual change. In the two cases presented, this change was positive and enriching for the patients and their loved ones. It is possible that reflecting on this change during the interview was beneficial and contributed to the results noted above.

More research is needed to evaluate the efficacy of the spiritual life review process described in this pilot study. Future protocols should consider larger sample sizes, randomized designs, and earlier follow-up, especially for those with advanced disease. Adaptation of the interview process and SLD development for other patient groups and those beyond the United States could provide benefit for participants and interesting and educational results. Clinically, sharing parts of a patient’s SLD with the health care team could enrich and deepen the bond between them and guide medical care in ways that champion the dignity of each person and the spirit alive within.

## Abbreviations:

Brief RCOPE: brief religious coping scale;
FACIT-Sp-12: functional assessment of chronic illness therapy–SWB;
LASA: linear analog self-assessment;
QOL: quality of life
SWB: spiritual well-being.
